# Designing a Novel Wide Bandgap Small Molecule Guest for Enhanced Stability and Morphology Mediation in Ternary Organic Solar Cells with over 19.3% Efficiency

**DOI:** 10.1002/advs.202401313

**Published:** 2024-04-03

**Authors:** Chenyang Zhang, Xiuzun Zhong, Xiaokang Sun, Jie Lv, Yaxiong Ji, Jiehao Fu, Chaoyue Zhao, Yiguo Yao, Guangye Zhang, Wanyuan Deng, Kai Wang, Gang Li, Hanlin Hu

**Affiliations:** ^1^ Hoffmann Institute of Advanced Materials Shenzhen Polytechnic University Shenzhen Guangdong 518055 China; ^2^ Institute of Flexible Electronics (IFE) Northwestern Polytechnical University Xi'an Shaanxi 710072 China; ^3^ School of Materials Science and Engineering Xiangtan University Xiangtan Hunan 411105 China; ^4^ Tsinghua Shenzhen International Graduate School Tsinghua University Shenzhen Guangdong 518055 China; ^5^ Department of Electronic and Information Engineering Research Institute for Smart Energy (RISE) The Hong Kong Polytechnic University Hong Kong Kowloon 999077 China; ^6^ College of New Materials and New Energies Shenzhen Technology University Shenzhen Guangdong 518118 China; ^7^ Institute of Polymer Optoelectronic Materials and Devices, State Key Laboratory of Luminescent Materials and Devices South China University of Technology Guangzhou Guangdong 510641 China

**Keywords:** charge management, crystallinity, phase separation, ternary organic solar cells

## Abstract

In this study, a novel wide‐bandgap small molecule guest material, ITOA, designed and synthesized for fabricating efficient ternary organic solar cells (OSCs) ITOA complements the absorbance of the PM6:Y6 binary system, exhibiting strong crystallinity and modest miscibility. ITOA optimizes the morphology by promoting intensive molecular packing, reducing domain size, and establishing a preferred vertical phase distribution. These features contribute to improved and well‐balanced charge transport, suppressed carrier recombination, and efficient exciton dissociation. Consequently, a significantly enhanced efficiency of 18.62% for the ternary device is achieved, accompanied by increased short‐circuit current density (*J_SC_*), fill factor (FF), and open‐circuit voltage (*V_OC_*). Building on this success, replacing Y6 with BTP‐eC9 leads to an outstanding PCE of 19.33% for the ternary OSCs. Notably, the introduction of ITOA expedites the formation of the optimized morphology, resulting in an impressive PCE of 18.04% for the ternary device without any postprocessing. Moreover, the ternary device exhibits enhanced operational stability under maximum power point (MPP) tracking. This comprehensive study demonstrates that a rationally designed guest molecule can optimize morphology, reduce energy loss, and streamline the fabrication process, essential for achieving high efficiency and stability in OSCs, paving the way for practical commercial applications.

## Graphical abstract

In this study, the design and synthesis of a novel wide‐bandgap small molecule guest, ITOA is presented. Utilizing ITOA as a morphology modulator, ternary organic solar cells achieved a remarkable power conversion efficiency (PCE) exceeding 19.3% along with exceptional stability. Impressively, without any postprocessing, the PCE of ternary devices reached an impressive 18.04%.

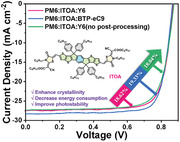

## 1 Introduction

In recent decades, the scientific community has increasingly focused on organic solar cells (OSCs) due to their potential to offer lightweight, flexible, and semi‐transparency photovoltaic solutions.^[^ ^1^, ^2^, ^3^, ^4^, ^5^, ^6^ ^]^ Central to organic solar cells (OSCs) is a bulk heterojunction (BHJ), encompassing p‐type (donors) and n‐type (acceptors) organic semiconductors.^[^ ^7^, ^8^, ^9^, ^10^ ^]^ Due to the emergence of Y‐series acceptors and novel molecules, coupled with the continuous refinement of device engineering, organic photovoltaics have made significant progress in recent years.^[^ ^11^, ^12^, ^13^ ^]^ Although OSCs have achieved power conversion efficiency (PCE) exceeding 19%, challenges related to device stability and large‐scale fabrication persist.^[^ ^14^, ^15^, ^16^, ^17^, ^18^, ^19^, ^20^, ^21^ ^]^ Recent literature has shed light on incorporating the third component as a viable strategy to enhance both the PCE and stability, placing ternary OSCs under an intensified spotlight.^[^ ^22^, ^23^, ^24^ ^]^

Ternary OSCs have been shown to possess an extended absorbance spectral range and a tunable energy level cascade. Such features are pivotal in enhancing the efficiency of solar photon utilization and charge transport dynamics.^[^ ^25^, ^26^ ^]^ However, a delicate interplay exists between phase separation and domain purity. Striking this balance remains a persistent challenge, requiring effective dissociation of excitons at the donor–acceptor interface but also ensuring seamless charge transport through unadulterated phases.^[^ ^27^, ^28^, ^29^ ^]^ Yet, when introduced into binary systems, only a few third components enhance the device performance. An inappropriate third component may act as the recombination center or a morphological trap, resulting in inferior device performance.^[^ ^30^ ^]^ Therefore, the designing and selecting of the appropriate third component with cascade energy level alignment, complementary absorbance coverage, and high crystalline properties are critical for building highly efficient ternary OSCs.

Up to date, PM6: Y‐series non‐fullerene acceptors (NFAs) represent the most commonly used binary hosts for ternary OSCs. This is partly due to their commercial availability and superior properties.^[^ ^31^, ^32^ ^]^ Tremendous effort has been devoted to innovating NFA third component because of their complementary absorbance spectra, commendable phase compatibility, and easier energy level adjustment. For example, Sun et al. proposed a facile strategy for the third component by sharing a common core unit with the host acceptor and a singular fluorinated end group of BTP‐F, Y6‐F, and L8‐BO‐F. The optimized ternary OSCs composed of PM6: BTP‐eC9: BTP‐F achieved an impressive PCE of 18.45%.^[^ ^33^ ^]^ Chen et al. designed asymmetric acceptors with varied end groups (BTP‐S10). Leveraging their phase compatibility with the host acceptor (L8‐BO), the ternary device achieved a remarkable PCE of 19.26%.^[^ ^34^ ^]^ Although various NFAs have been explored in high‐performance ternary OSCs, the design principles of the third component remain unclarified, and most of the third components were initially explored as donors/acceptors in binary blends.

In the early stage, the design architecture of the third component focused on achieving complementary absorbance, cascade energy level stratification, and improved carrier behavior. Yet, with the advent of superior‐performing binary systems, several notably efficient ternary OSCs have been documented, despite encountering suboptimal energy levels and crystalline properties in their third component. For instance, Wei et al. introduced a small molecule donor BTID‐2F with an unfavorable energy level arrangement into the binary system, resulting in a relatively small energy disorder in the ternary component, which improved *V_OC_*.^[^ ^35^ ^]^ As a result, a PCE of 17.98% was achieved for PM6:Y6‐based ternary device. Ma et al. reduced the crystallinity of the ternary film by introducing a third component to achieve a more balanced charge carrier mobility.^[^ ^36^ ^]^ The ternary device, processed by blade‐coating, showed an improved efficiency of 12.02%. Therefore, the design principle of the third component should be reconsidered, and morphological optimization and energetic ordering of the host blend film are becoming more and more important, even though the morphological control of the ternary blend film has proven elusive. Furthermore, conventional OSCs often necessitated the use of high‐boiling point additives or post‐treatments such as thermal annealing and solvent vapor annealing to achieve optimal efficiency. However, additives were undesirable in large‐scale industrial printing processes, and post‐treatments also escalated production costs. Therefore, there was considerable interest in high‐performance as‐cast devices.^[^ ^37^, ^38^, ^39^ ^]^ Yan et al. achieved efficient as‐cast devices through a ternary strategy, yielding a PCE of 16.68%, thereby providing insights and guidance for high‐performance as‐cast OSCs.^[^ ^40^ ^]^

In our previous studies, we focused on the side chains of the central core and reported the isomerization effect of chlorine‐substitution positions on small molecule guest materials. Varying the position of the halogen atom could slightly affect the molecular packing property, and the ternary devices based on the α‐Cl substituted guest achieved a PCE of 18.96%.^[^ ^41^ ^]^ We also explored end‐group engineering in small molecule guests by adopting cyanooctyl ester and esterified rhodamine as end groups. Both small molecule guest‐based ternary devices performed well and achieved a PCE of 18.11%.^[^ ^42^ ^]^ In this contribution, we designed and synthesized a novel wide‐bandgap small molecule denoted as ITOA. It features a fused‐ring indacenodithienothiophene (IDTT) as the central core and octyl (E)‐2‐cyano‐2‐(3‐octyl‐4‐oxothiazolidin‐2‐ylidene) acetate as terminal end groups. The central IDTT core ensures the conjugated rigidity and tight interchain π–π overlap. The modest electron‐withdrawing end group, along with long alkyl chains, allows for adjustments in absorbance, energy level, and miscibility. This molecule exhibits a lower highest occupied molecular orbital (HOMO) level than PM6, complementary absorbance with PM6:Y6 system in the short‐wavelength region, and strong crystallinity. Comprehensive morphology studies demonstrate that the introduction of ITOA enhances the crystallinity, resulting in more favorable vertical phase distribution and phase separation. As a result, ternary OSCs exhibited reduced energy loss, suppressed carrier recombination, and enhanced and more balanced charge transport. Specifically, the PM6: ITOA: Y6 ternary device achieved a remarkable PCE of 18.62% with enhanced *V_OC_* (0.870 V), *J_SC_* (27.57 mA cm^−2^) and FF (77.63%). Replacing Y6 with BTP‐eC9 delivered an impressive PCE of 19.33%, a record value of PM6: BTP‐eC9‐based ternary systems, indicating the universality of ITOA. Interestingly, the incorporation of ITOA accelerates the film formation of the optimal morphology without any postprocessing. Nonannealed PM6:Y6‐based ternary OSCs obtained a PCE of 18.04%, which represents among the most elevated values reported for devices without post‐processing. Additionally, in N_2_ atmosphere the ternary device exhibited significantly extended operational stability under maximum power point (MPP) tracking, with T_80_ recorded at 144 h.

## 2 Results and Discussion

### 2.1 Synthesis and Properties

**Figure 1:**
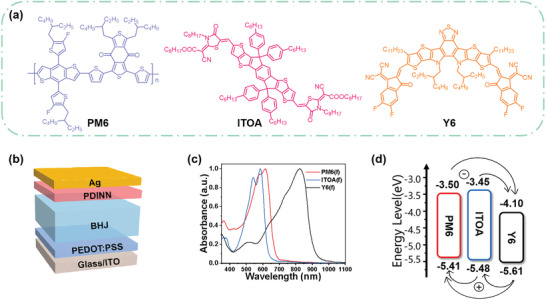
a) Chemical structures of PM6, ITOA, and Y6. b) Illustration of the OSCs device configuration. c) Normalized UV–vis absorbance spectra of pure films, individually. d) Energy level evaluation for PM6, ITOA, and Y6.

A mixture of IDTT‐CHO (0.108 g, 0.1 mmol), octyl (E)‐2‐cyano‐2‐(3‐octyl‐4‐oxothiazolidin‐2‐ylidene)acetate (0.409 g, 1.0 mmol) was subjected to three vacuum/ nitrogen cycles, and degassed CHCl_3_ (30 mL) and piperidine (0.5 mL) was added to the flask. The resulting solution was stirred and refluxed for 12 h. The solvent was removed and the residue was subjected to column chromatography using hexanes/CH_2_Cl_2_ (2/1) as the eluent. The crude solid was purified and adjust the state from methanol and CHCl_3_ mixture was three times to afford ITOA as a red solid (0.097 g, 52%). Detailed ^1^H NMR, ^13^C NMR, and HRMS data confirming the precise chemical structure of ITOA are provided in Figures S2–S4 (Supporting Information). Additionally, thermal gravimetric analysis (TGA) of ITOA (Figure S5, Supporting Information) revealed a decomposition onset at 367 °C, accompanied by a circa 5% reduction in mass, highlighting its thermal stability at current operating temperatures. As shown in **Figure** **1**a, the chemical structures of PM6, Y6, and ITOA are depicted, with PM6 and Y6 purchased from Organtec. Ltd. In Figure 1b, the traditional device structure of OSCs (ITO/PEDOT:PSS/BHJ/PDINN/Ag) was employed. The UV–vis absorbance measurements revealed that maximum absorbance peaks were at 615 nm for PM6 and 825 nm for Y6 respectively, as shown in Figure 1c. In contrast, ITOA exhibited absorbance in a shorter wavelength region with a strong shoulder at 541 nm and its maximum absorbance peak at 584 nm. We also performed UV–vis absorbance measurements on the blend film (Figure S6, Supporting Information), and we found that the ternary blend film showed a wider absorption range than the binary film. In order to further explore the effect of ITOA on the absorbance of the active layer film, the results showed that the PM6:ITOA:Y6 blend film had a higher absorption coefficient than the binary film (Figure S7, Supporting Information), which improved the light collection ability of the film. This indicated the extended absorbance range and enhanced absorbance intensity in the blend film, which could potentially lead to a larger *J_SC_*. The optical bandgap of ITOA to be ≈1.99 eV was calculated using the formula of *E*_g_^opt^ = 1240/*λ*_onset_. To assess the accuracy of the optical bandgap. The cyclic voltammetry (CV) measurements for ITOA were employed (Figure S8, Supporting Information), and using the formula of *E*_HOMO/LUMO_ = −*E* (*E*_onset_, _ox/red_ +4.80−*E*_Fc/Fc+_) (eV) calculated the electrochemical bandgap. We yielded the lowest unoccupied molecular orbital (LUMO) and HOMO for ITOA were −3.45 and −5.48 eV, respectively (Table S1, Supporting Information). Furthermore, density functional theory (DFT) calculations were applied at the B3LYP/6‐31G (d, p) level in Figure S9 (Supporting Information), following the same trend as the LUMO and HOMO energy levels obtained from the electrochemical tests.^[^ ^43^ ^]^ Finally, the energy level evaluation of PM6, Y6, and ITOA is illustrated (Figure 1d). In addition, the torsion angle is only 0.82° between the intermediate nucleus (IDTT) and the end group in Figure S9 (Supporting Information), indicating that ITOA possesses superior planarity, which promotes close packing and charge transfer of π–π between molecules.

### 2.2 Device Characterization and Performance

**Figure 2:**
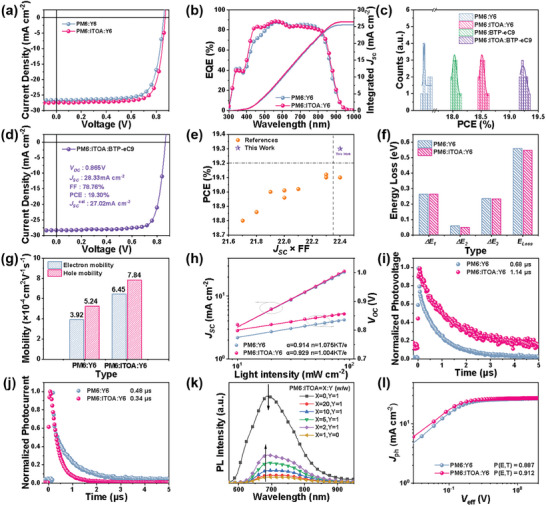
Device performance and characterization. a) The *J–V* traces and b) EQE spectra for PM6:Y6 and PM6: ITOA: Y6 systems. c) PCE distributions across different device types. d) The *J–V* profile of the optimally blended PM6: ITOA: BTP‐eC9 system. e) Comparative analysis between previously reported high‐efficiency PM6: BTP‐eC9‐based ternary OSCs with PCEs surpassing 18.8% and the current study. Assessments of f) Energy loss, g) SCLC, h) Light‐intensity dependence, i) TPV, and j) TPC for the PM6:Y6 and optimal PM6: ITOA: Y6 systems. k) PL spectra for pure PM6, ITOA, and the composite PM6: ITOA films. l) *J*_ph_–*V*_eff_ traces for binary and ternary OSCs.

All the devices were fabricated using a spin‐coating method, with 0.5 vol% 1‐Chloronaphthalene (1‐CN) as an additive in chloroform solution employed, maintaining a total host concentration of 13.8 mg mL^−1^. The mass ratio of the D/A (donor to acceptor) was configured at PM6:Y6 = 1:1.2 in the binary devices. Upon the introduction of ITOA, the optimal mass ratio for the ternary devices was determined to be 1:0.05:1.2. Under standard AM 1.5G illumination with 100 mW cm^−2^ intensity, all devices were subjected to testing in an air environment. In **Table** **1** and **Figure** **2**a, the resulting photovoltaic parameters and current–voltage (*J–V*) curves are shown, respectively. Device optimization predominantly entailed the fine‐tuning of guest donor proportions and the calibration of annealing temperatures. As shown in Tables S2 and S3 (Supporting Information), comprehensive optimization findings can be found. The PM6:Y6 binary device showcased a *V_OC_* of 0.859 V, a *J_SC_* of 26.60 mA cm^−2^, and an FF of 73.81%, resulting in a PCE of 16.87% that aligns with the values reported in the literature.^[^ ^44^ ^]^ In a comparative analysis with binary devices, the superior performance of ternary devices, marked by an enhancement in the *V_OC_* of 11 mV, can be primarily ascribed to the reduction in energy loss inherent. The *J_SC_* of the ternary devices was noted to be 27.57 mA cm^−2^, substantially higher than that of the binary devices (26.60 mA cm^−2^). This enhancement could be attributed to the improved carrier transport capability and the increased absorbance of ITOA in the short wavelength of the ternary blend layer. As depicted in Figure 2b, the ternary devices manifested an augmented photoresponse in the short wavelength 400–500 nm region, as evidenced by the external quantum efficiency (EQE) spectra, further substantiating the contribution from ITOA. From the EQE spectra integration, photocurrent values were discerned to be 25.50 and 26.54 mA cm^−2^ for binary and ternary OSCs, respectively, which agreed with the discussed *J_SC_* values (Table 1) with an error within 5%. Likewise, the introduction of 5 wt.% ITOA led to an optimized morphology of the ternary blend film, elevating the FF value from 73.81% to 77.63%. Conclusively, the ternary device PM6: ITOA (5 wt.%): Y6 delivered an outstanding PCE of 18.62%, ranking among the paramount values reported thus far for PM6:Y6‐based ternary devices (Table S4, Supporting Information). In light of the outstanding photovoltaic performance of ITOA, we additionally introduced it as the third component into the PM6:BTP‐eC9 system. Delightfully, akin to the performance in the PM6:Y6 system, enhancements in *V_OC_*, *J_SC_*, and FF were noted, resulting in an outstanding PCE of 19.33% (Figure 2d), which is the highest value reported thus far for PM6: BTP‐eC9‐based ternary systems (Figure 2e; Table S5, Supporting Information).

**Table 1:** Performance metrics for binary and optimally engineered ternary devices.

Active layer	*V_OC_* [V]	*J_SC_* [mA cm^−2^]	FF [%]	PCE(PCE_ave_ ^a^) [%]
PM6:Y6	0.859	26.60	73.81	16.87 (16.81 ± 0.04)
PM6:ITOA:Y6	0.870	27.57	77.63	18.62 (18.47 ± 0.09)
PM6:BTP‐eC9	0.853	27.81	76.41	18.12 (18.10 ± 0.04)
PM6:ITOA:BTP‐eC9	0.866	28.33	78.79	19.33 (19.20 ± 0.12)

^a)^ Values represent averages derived from ten distinct batches.

In response to the observed elevation of *V_OC_* in the ternary devices, we sought to explore the impact of ITOA incorporation on the device's energy loss. To this end, both binary and ternary OSCs underwent Electroluminescence (EL) spectral and Fourier Transform Photocurrent Spectroscopy‐External Quantum Efficiency (FTPS‐EQE) analyses, as illustrated in Figure S12 (Supporting Information). The energy losses can be quantified using the formula Δ *E*_loss_ =  Δ*E*_1_ + Δ*E*_2_ + Δ*E*_3_.^[^ ^45^ ^]^ ∆*E*_1_ represents the inevitable above‐bandgap absorbance radiative recombination, typically ranging from 0.25 to 0.30 eV. ∆*E*_2_ refers to the below‐bandgap radiative recombination, determined by the charge transfer (CT) energy, recombination energy, or energetic disorder affecting sub‐bandgap absorbance in the optically active blend film. The reduction of ∆*E*_2_ can lead to the generation of higher voltages.^[^ ^46^ ^]^ As for ∆*E*_3_, it is closely related to electroluminescence efficiency: Δ *E*_3_ =   − *kT*ln(*EQE*_EL_).^[^ ^47^ ^]^ In our experimental results (Figure 2f, detailed parameters in **Table** **2**), we found that both binary and ternary devices exhibited the same *∆E*_1_ (0.264 eV) and the basic equivalent of *∆E*_3_ (0.236 eV for binary OSCs, 0.233 eV for ternary OSCs). The smaller energy loss in the ternary devices can mainly be attributed to lower values of *∆E*_2_. This may be due to the enhanced crystallinity of the PM6: ITOA: Y6 blend film, which reduces the energy disorder of the ternary OSCs, and finally shows lower Ulbach energy (24.2 meV) and ∆*E*_2_ (0.049 eV) values than the binary OSCs (25.0 meV and 0.060 eV, respectively) in Figure S12c (Supporting Information).^[^ ^48^ ^]^

**Table 2:** Energy loss metrics for binary and optimally engineered ternary OSCs.

Active layer	E_g_[eV]	q*V*_SQ,rad_[eV]	q*V*_rad_[eV]	EQE_EL_[%]	*ΔE*_1_[eV]	*ΔE*_2_[eV]	*ΔE*_3_[eV]	*E*_loss_[eV]
PM6:Y6	1.42	1.156	1.096	1.05E‐02	0.264	0.060	0.236	0.560
PM6:ITOA:Y6	1.42	1.156	1.107	1.18E‐02	0.264	0.049	0.233	0.546

To elucidate the observed augmentation in FF and *J_SC_* characteristics, we embarked on a thorough examination of carrier behavior mechanisms within both binary and ternary setups. Primarily, we explored the charge transport dynamics via the space charge limited current (SCLC) methodology. The hole‐only and the electron‐only devices were constructed adopting ITO/PEDOT:PSS/BHJ/MoO_3_/Ag and ITO/ZnO/BHJ/PDINN/Ag, respectively. Referencing Figure 2g and Table S6 (Supporting Information), it becomes evident that the hole (*µ*_h_) and electron (*µ*_e_) mobilities for the PM6:Y6, manifest values of 5.24 × 10^−4^ and 3.92×10^−4^ cm^2^ V^−1^ s^−1^, respectively. The ITOA‐incorporated ternary system evinced a conspicuous surge in both *µ*_h_ (7.84 × 10^−4^ cm^2^ V^−1^ s^−1^) and *µ*_e_ (6.45 × 10^−4^ cm^2^ V^−1^ s^−1^). Further scrutiny revealed that the *µ*_h_/*µ*_e_ ratio underwent a decrement from 1.33 in the binary paradigm to 1.21 in the ITOA‐based ternary configuration, a phenomenon that aligns congruously with its superior FF and *J_SC_* metrics observed in ternary OSCs.^[^ ^49^, ^50^ ^]^

Besides, the study of bimolecular recombination and trap‐assisted mechanisms was executed through the analysis of light‐intensity dependence measurements. In Figure 2h, the correlations of *J_SC_* and *V_OC_* as functions of light intensity (*P*_light_) are graphically represented. From a quantitative perspective, the linear fit for *J_SC_* in relation to *J*_SC_ ∝ *P*_light_^α^ produced a slope (α) that exceeded 0.90. In the analysis of the ternary device, the derived slope value (α) from the linear regression was determined to be 0.929. This value approaches unity, suggesting a marginal decrease in bimolecular recombination relative to the binary device, which exhibited an α value of 0.914.^[^ ^49^ ^]^ In a parallel analysis, the relationship between *V_OC_* and *P*_light_ can be described by the equation *V*_OC_∝(*nkT*/e) ln(*P*_light_). Herein, *k* represents the Boltzmann constant, *T* stands for the absolute temperature, and e denotes the elemental charge. Typically, an n parameter greater than 1 signifies the presence of trap‐assisted recombination. Our fitted results demonstrate *n* values of 1.075 for the binary device and a notably lower 1.004 for the ternary device. This suggests an absence of significant trap‐assisted recombination in these configurations. Notably, the ternary system exhibits a subdued n value, indicating a reduction in trap‐assisted recombination upon the inclusion of ITOA as a third constituent.^[^ ^51^ ^]^ These findings align with the noted improvement in FF and the overall device performance. In addition to light‐intensity dependence measurements, transient photovoltage (TPV) and transient photocurrent (TPC) tests were conducted to assess the charge recombination and extraction capabilities of the devices (Figure 2i,j). Employing an exponential decay fitting method, the PM6: ITOA: Y6 devices exhibited an extended carrier lifetime (1.14 µs) compared to the PM6:Y6 devices (0.68 µs) and a reduced charge extraction time (0.48 µs for binary device and 0.34 µs for ternary device, respectively). These findings suggest that the ternary devices suppressed trap‐assisted recombination, facilitating faster charge extraction and resulting in enhanced FF and *J_SC_* values.^[^ ^52^, ^53^ ^]^

Figure 2k displays the photoluminescence (PL) spectra of pure PM6, ITOA, and PM6: ITOA composite films at varying mass ratios. PM6 and ITOA exhibited emission peaks at 685 and 690 nm, respectively. As the ITOA content surged, the emission intensity of the PM6: ITOA blend films progressively intensified. Intriguingly, the emission peak transitions increasingly from PM6 toward ITOA, hinting at the underlying energy transfer between the two materials.^[^ ^54^ ^]^ To delve deeper into the role of ITOA in exciton dissociation, we charted the interplay between photocurrent density (*J*_ph_) and effective voltage (*V*_eff_) as presented in Figure 2l. The exciton dissociation probability, P(E,T), serves as a pivotal metric for elucidating exciton dissociation and charge extraction dynamics, with a detailed calculation protocol meticulously expounded in the Supporting Information. Remarkably, the PM6: ITOA: Y6 devices boast the P (E, T) value of 91.2%, outperforming the PM6:Y6 device at 88.7%. This enhancement underscores an augmented exciton dissociation and charge extraction in the PM6: ITOA: Y6 devices, culminating in improved FF and *J_SC_*.^[^ ^55^ ^]^

### 2.3 Morphological and Structural Analyses of Blended Films

**Figure 3:**
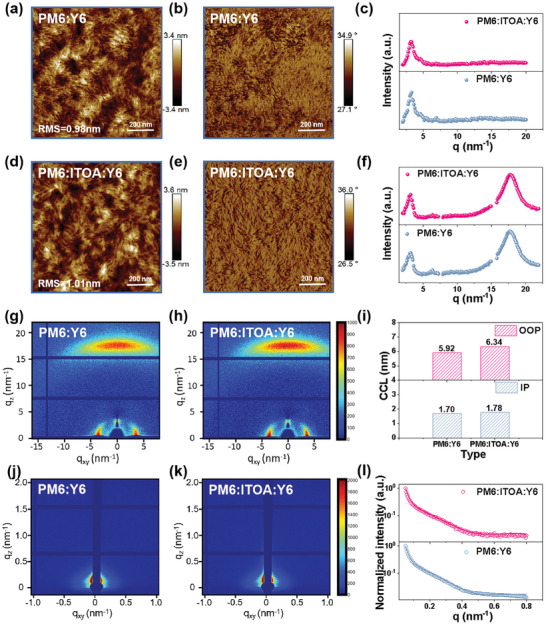
Morphological and structural analyses of blended films. a,d,g,j) AFM height, phase, GIWAXS, and GISAXS images respectively for binary blends. b,e,h,k) Corresponding characterizations for ternary blends. GIWAXS intensity delineations along c) the (100) and f) the (010) planes. i) Evolution in the CCL for both IP and OOP diffraction peaks of binary and ternary films. l) IP scattering trajectories extracted from GISAXS patterns of both blend types.

To critically assess the surface morphology and quality of binary and the optimized ternary thin films, we employed Atomic Force Microscopy (AFM). The height images of these optimized films are depicted in **Figure** **3**a,b. The root‐mean‐square (RMS) values for these films were found to be proximal to 1 nm, ensuring excellent contact of the film with the top interface. Morphological inspection from the phase images (Figure 3d,e) revealed the shaping of a nano‐interpenetrating dual‐fiber network, conducive to charge transport and exciton dissociation, ultimately paving the way for elevated FF and *J_SC_* values.^[^ ^56^ ^]^

To delve deeper into the crystallinity variations of these films, we performed rigorous grazing incidence wide/small‐angle X‐ray scattering (GIWAXS/GISAXS) characterizations on both binary and the optimized ternary films in Figure 3g,h,j,k. As shown in Figure 3c,f, integration of the two‐dimensional plots of GIWAXS along the (100) and (010) directions revealed scattering profiles. Both PM6:Y6 and PM6: ITOA: Y6 films showed scattering peaks in the in‐plane (IP) and out‐of‐plane (OOP) directions, predominantly favoring the OOP orientation. In the OOP direction, the scattering peak position for the ternary film was pinpointed ≈16.68 nm^−1^, which is markedly larger than its binary counterpart (16.64 nm^−1^). Based on the formula D = 2π/Q, we deduced a tighter π–π stacking in the OOP direction for the ternary film, promising enhanced charge transport.^[^ ^57^, ^58^ ^]^ Furthermore, the intensity of the scattering peak for the ternary film in the OOP direction noticeably surpassed that of the binary film. The CCL serves as a pivotal metric to evaluate material crystallinity. Employing relevant calculations, the CCL values (elaborated details in Table S7, Supporting Information; Figure 3i) for binary and ternary films in the OOP direction were discerned to be 1.70 and 1.78 nm, respectively. This underscores the superior crystallinity of the ternary film, which resonates seamlessly with the RMS value analysis. The scattering profiles obtained from GISAXS patterns are fitted with a universal model based on the effective interface approximation of the distorted wave Born approximation (DWBA), as demonstrated in Figure 3l.^[^ ^59^ ^]^ Our computational findings reveal an amorphous phase (ξ) domain size of 34.9 nm for the binary film, contrasted with a slightly reduced to 32.2 nm for the ternary film. This accentuates that the introduction of ITOA engenders an increase in the phase separation scale of the film, subsequently yielding an elevation in *J_SC_* and FF values. This observation harmoniously resonates with the outcomes derived from morphological and crystallinity analyses. In addition, the contact angles of pure PM6, ITOA, and Y6 films with water and ethylene glycol were evaluated in Figure S14 (Supporting Information), with detailed parameters and results delineated in Table S8 (Supporting Information). Subsequent computations revealed surface‐free energies of 26.79 mN m^−1^ for PM6, 21.08 mN m^−1^ for ITOA and 30.77 mN m^−1^ for Y6. The Flory‐Huggins interaction parameter (*χ*) quantitatively characterizes material miscibility—smaller *χ* values denote enhanced miscibility.^[^ ^60^ ^]^ Based on our calculations, the χ_PM6: ITOA_ value (0.34*K*) is lower than the χ_Y6: ITOA_ value (0.91*K*), underlining the superior blend compatibility of ITOA with PM6.

**Figure 4:**
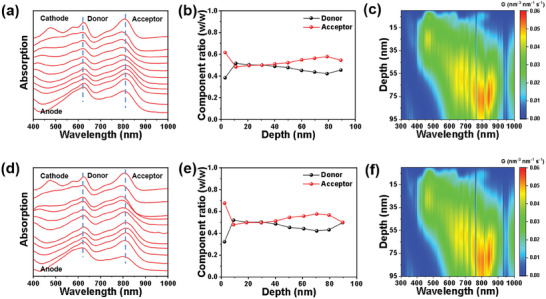
Depth‐dependent properties and simulations of blended films. a,b,c) FLAS, compositional variation profiles, and simulated exciton generation rates, respectively, for binary films. d,e,f) Corresponding analyses for ternary films.

Furthermore, film‐depth‐resolved light absorbance spectroscopy (FLAS) was employed to investigate the vertical phase distribution.^[^ ^61^, ^62^ ^]^ **Figure** **4**a,d reveal absorbance peaks at ≈ 610 and 820 nm, attributable to PM6 and Y6 of the host system. An intensified absorbance ≈580 nm, adjacent to the substrate, aligns with absorbance traits of ITOA, hinting at its concentration in sub‐layers near the anode (ITO/PEDOT:PSS). Sub‐layer spectra provide insights into the donor/acceptor ratio in this zone, with specifics in the supporting information. Figure 4b highlights a prevalent distribution of the acceptor near the anode, potentially hindering charge dynamics. Figure 4e underscores the integration of ITOA‐mediated distribution of the acceptor near the anode and leads to a donor‐riched region near the anode and an acceptor‐riched region near the cathode. This ordered vertical phase distribution could facilitate the charge transport and reduce the carrier recombination.^[^ ^63^ ^]^ Additionally, post‐processing FLAS using the optical matrix transfer model facilitates the simulated assessment of exciton generation rate's vertical distribution. As portrayed in Figure 4c,f, donor/acceptor predominantly generates excitons near the cathode (PDINN) and anode, complicating charge transport. Yet, the augmented crystallinity of ternary films combined with its nano‐interpenetrating dual‐fiber network simplifies this, leading to enhanced FF and *J_SC_* values in PM6: ITOA: Y6 devices.

### 2.4 Characterization and Performance Assessment of Films and Devices Without Postprocessing

**Figure 5:**
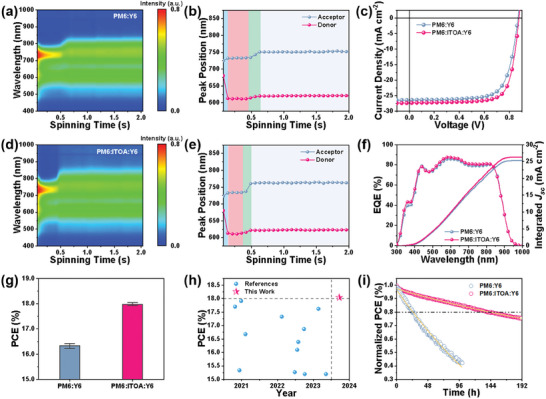
Characterization and performance assessment of films and devices without post‐processing. a,d) In‐situ UV–vis absorbance during the spin‐coating process for binary and the optimized ternary films. b,e) Peak positioning for donor and acceptor in PM6:Y6 and PM6: ITOA: Y6 films, respectively. c) The *J*–*V* traces and f) EQE spectra for the PM6: Y6 and PM6: ITOA: Y6 systems. g) PCE distributions across different device types. h) Comparative evaluation between previously reported high‐efficient devices without postprocessing boasting PCEs above 15% and the current investigation. i) MMP light stability assessment under a N_2_ environment.

To elucidate the crystallinity dynamics inherent in the blend films, we utilized in situ UV–vis spectroscopy to unravel the underlying drying mechanism (**Figure** **5**a,d). Figure 5b,e displayed contour plots of the absorbance spectra for binary and ternary films during drying, respectively. Within the initial 2s of film formation, we discerned roughly four phases: transition from liquid to solid, film thinning under centrifugal forces, spontaneous aggregation of the acceptor, and film solidification. The introduction of ITOA expedited the film formation, particularly impacting the thinning duration and Y6 aggregation. This phenomenon was conducive to the formation of a high‐crystallinity pure acceptor phase, which helped to enhance charge transport.^[^ ^64^ ^]^ In addition, the position of the receptor peak in the ternary system was slightly redshifted, which made it possible to obtain a high *J_SC_*. In essence, the ternary films exhibit heightened crystallinity and optimal phase separation dimensions. To further validate these findings, photovoltaic performance characterizations of nonannealed devices are shown in Figure 5c,f,g. Notably, the ternary device achieved a PCE of 18.04%, distinctly surpassing its binary counterpart, setting the record for no postprocessing OSCs (Figure 5h; Table S9, Supporting Information). Stability remains a focal area of research.^[^ ^65^ ^]^ Under N_2_ atmosphere and continuous illumination at 100 mW cm^−2^, maximum power point (MPP) tracking was undertaken. As portrayed in Figure 5i, the binary device retained only 80% of its initial PCE post 25 h, whereas the T_80_ of ternary devices was recorded at 144 h. The improved light stability under N_2_ atmosphere can be attributed to the enhanced crystallinity of the ternary blend films.^[^ ^66^, ^67^ ^]^ This insinuates the potential of ITOA in augmenting the photostability of OSCs.

## 3 Conclusion

The introduction of ITOA, a novel A‐D‐A type molecule, into PM6:Y6 binary system has demonstrated considerable promise in enhancing both photovoltaic performance and device stability. The GIWAXS/GISAXS and FLAS characterizations have unveiled the role of ITOA in improving blend crystallinity, optimizing vertical phase distribution, and expanding the absorbance spectrum of ternary systems. Consequently, ternary OSCs have demonstrated improvements in energy loss, charge carrier transport, and suppressed charge carrier recombination behaviors, leading to enhanced values of *V_OC_*, *J_SC_*, and FF. Notably, ternary devices incorporated with ITOA achieved a commendable PCE of 18.62% in PM6:Y6 system and 19.33% in PM6: BTP‐eC9 system, one of the highest values currently reported for their respective types. Simultaneously, ITOA‐based ternary devices, without postprocessing, achieved a PCE of 18.04%, establishing a new benchmark for OSCs and a mitigation strategy for commercial production. Additionally, stability assessments underscored the potential of ITOA in enhancing the longevity of the ternary devices. These findings highlight the role of careful material selection and design in advancing OSCs technology. It provides a new scheme for the molecular design of the third component with an aim to achieve reduced energy loss, efficient charge transport, and reasonable morphology, and ultimately obtaining highly efficient and stable ternary OSCs.

## Conflict of Interest

The authors declare no conflict of interest.

## Supporting information

Supporting Information

## Data Availability

Research data are not shared.
