# Supplementary material for: Designing a Novel Wide Bandgap Small Molecule Guest for Enhanced Stability and Morphology Mediation in Ternary Organic Solar Cells with over 19.3% Efficiency
Source: Adv Sci (Weinh). 2024 Apr 3;11(23):2401313. doi: 10.1002/advs.202401313 (PMC11187928; doi:10.1002/advs.202401313)
Supplement: Supplementary file 1 — Supporting Information [file ADVS-11-2401313-s001.pdf]

ADVANCED SCIENCE
Open Access

Supporting Information

for *Adv. Sci.*, DOI 10.1002/advs.202401313

Designing a Novel Wide Bandgap Small Molecule Guest for Enhanced Stability and Morphology Mediation in Ternary Organic Solar Cells with over 19.3% Efficiency

Chenyang Zhang, Xiuzun Zhong, Xiaokang Sun, Jie Lv, Yaxiong Ji, Jiehao Fu, Chaoyue Zhao, Yiguo Yao, Guangye Zhang, Wanyuan Deng, Kai Wang, Gang Li* and Hanlin Hu**

Supporting Information

Designing A Novel Wide Bandgap Small Molecule Guest for Enhanced Stability and Morphology Mediation in Ternary Organic Solar Cells with over 19.3% Efficiency

Chenyang Zhang,[a],[b] Xiuzun Zhong,[b] Xiaokang Sun,[a],[c] Jie Lv,[a] Yaxiong Ji,[d] Jiehao Fu,[e] Chaoyue Zhao,[f] Yiguo Yao,[b] Guangye Zhang,[f] Wanyuan Deng,[g] Kai Wang,*[b] Gang Li,*[e] and Hanlin Hu*[a]

[a] C. Zhang, X. Sun, Dr. J. Lv, Prof. H. Hu
Hoffmann Institute of Advanced Materials, Shenzhen Polytechnic University
Guangdong, Shenzhen 518055 (China)
E-mail: hanlinhu@szpu.edu.cn

[b] C. Zhang, X. Zhong, Y. Yao, Prof. K. Wang
Institute of Flexible Electronics (IFE), Northwestern Polytechnical University
Shaanxi, Xi'an 710072 (China)
E-mail: kaiwang@nwpu.edu.cn

[c] X. Sun
School of Materials Science and Engineering, Xiangtan University
Hunan, Xiangtan 411105 (China)

[d] Y. Ji
Tsinghua Shenzhen International Graduate School, Tsinghua University
Guangdong, Shenzhen 518055 (China)

[e] J. Fu, Prof. G. Li
Department of Electronic and Information Engineering, Research Institute for Smart Energy (RISE), The Hong Kong Polytechnic University
Hong Kong, Kowloon 999077 (China)
E-mail: gang.w.li@polyu.edu.hk

[f] C. Zhao, Prof. G. Zhang
College of New Materials and New Energies, Shenzhen Technology University
Guangdong, Shenzhen 518118 (China)

[g] Dr. W. Deng
Institute of Polymer Optoelectronic Materials and Devices, State Key Laboratory of Luminescent Materials and Devices, South China University of Technology
Guangdong, Guangzhou 510641 (China)

1. General Experimental Details

Methods and Materials: All reagents from commercial sources were used without further purification. Solvents were dried and purified using standard techniques. Reactions were carried out under nitrogen atmosphere when appropriate. All compounds were characterized by NMR spectroscopy on Bruker Avance III Ultrashield Plus instruments using a 500 MHz proton frequency at the given temperatures. The spectra were referenced to the internal standard TMS. The photophysical and electrochemical properties of the materials were measured on UV-visible-near infrared spectrograph (Agilent Cary 60 spectrometer) and electrochemistry workstation (CHI660A, Chenhua Shanghai), respectively. The DSC samples were prepared by scraping and collecting the annealed blend films from glass, which were prepared by coating and drying the blend solutions. Atomic force microscopy (AFM) images were characterized with a Veeco Multi-Mode 8 in a tapping mode. GIWAXS and GISAXS measurements were carried out on beamline BL16B1 at the Shanghai Synchrotron Radiation Facility (SSRF). The light stability test was performed on an unpackaged device at an ambient temperature of 25±5 ℃ in a nitrogen atmosphere. Continuous tracking of the maximum power point under continuous illumination conditions in a solar simulator using an AM 1.5 filter with a power of 100mw cm^{-2}.

2. Synthetic Procedures

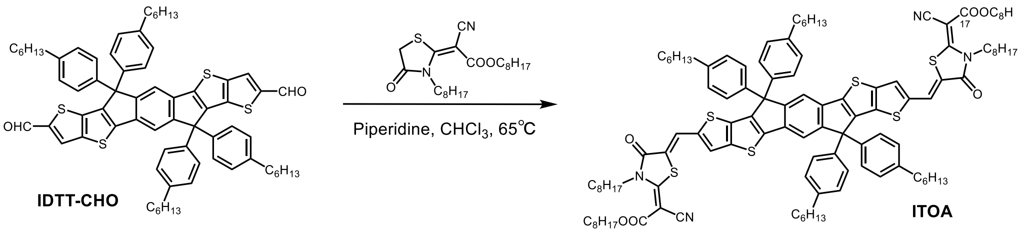

Figure S1 The synthetic route of ITOA.

Synthesis of ITOA: A mixture of IDTT-CHO (0.108 g, 0.1 mmol), octyl (E)-2-cyano-2-(3-octyl-4-oxothiazolidin-2-ylidene)acetate (0.409 g, 1.0 mmol) was subjected to three vacuum/ nitrogen cycles, and degassed $CHCl_3$ (30 mL) and piperidine (0.5 mL) was added to the flask. The resulting solution was stirred and refluxed for 12 h. The solvent was removed and the residue was subjected to column chromatography using hexanes/CH_2Cl_2 (2/1) as the eluent. The crude solid was purified and adjust the state from methanol and $CHCl_3$ mixture three times to afford ITOA as a red solid (0.097 g,

52%). ^{1}H NMR (500 MHz, $CDCl_3$): δ = 7.88(s, 2H), 7.68(s, 2H), 7.57(s, 2H), 7.18 (d, *J* = 8.3 Hz, 8H), 7.12 (d, *J* = 8.3 Hz, 8H), 4.36 (t, *J* = 7.8 Hz, 4H), 4.31 (t, *J* = 6.8 Hz, 4H), 2.57 (t, *J* = 8.0 Hz, 8H), 1.77 (q, *J* = 6.8 Hz, 8H), 1.60 (t, *J* = 7.8 Hz, 8H), 1.48 - 1.36 (m, 16H), 1.31 - 1.24 (m, 48H), 0.90 - 0.85 (m, 24H). ^{13}C NMR (126 MHz, $CDCl_3$): δ = 166.61, 165.59, 163.44, 154.50, 147.90, 146.64, 143.58, 142.33, 139.32, 139.06, 138.49, 136.38, 128.78, 128.10, 127.85, 126.58, 117.75, 116.41, 115.18, 75.87, 66.36, 63.13, 49.43, 45.46, 35.60, 31.80, 31.75, 31.70, 31.23, 29.19, 29.11, 28.92, 28.63, 25.94, 25.82, 22.65, 22.62, 22.59, 17.68, 14.08.

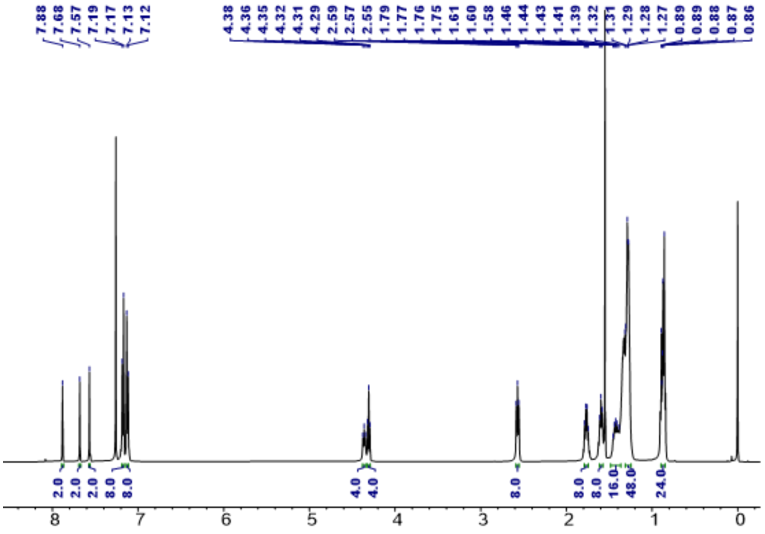

Figure S2 ^{1}H NMR spectrum of ITOA in $CDCl_3$.

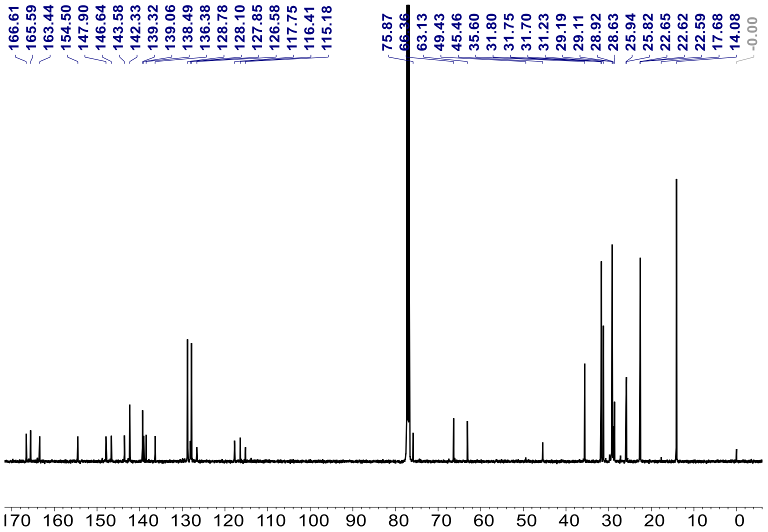

Figure S3 ^{13}C NMR spectrum of ITOA in $CDCl_3$.

3. High-resolution mass spectrometry (HRMS)

The High-resolution mass spectrometry (HRMS) data was recorded using Agilent 6550 iFunnel Q-TOF. HRMS (+APCI, m/z): calcd. for $C_{114}H_{142}N_4O_6S_6$ $[M+H]^+$: 1856.77; found 1856.83.

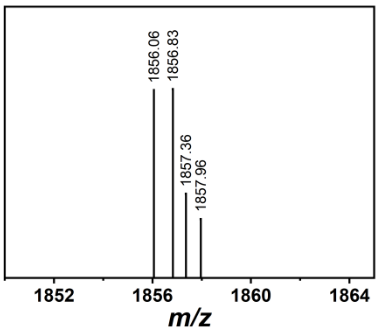

Figure S4. High-resolution mass spectrometry (HRMS) of ITOA.

4. Thermogravimetric analyses (TGA)

Thermogravimetric analyses (TGA) was performed with a TA TGA 50 under nitrogen atmosphere, with a set ramp rate of 10 °C/min, and using Al_2O_3 (alox) crucibles.

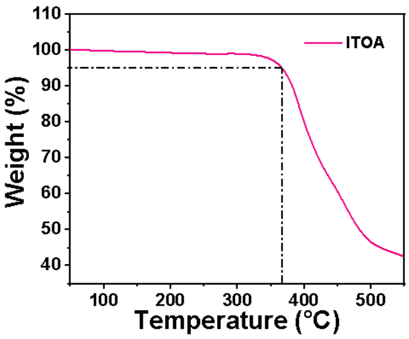

Figure S5 Thermogravimetric analyses (TGA) of ITOA.

5. UV-vis absorbance spectrum and absorption coefficients

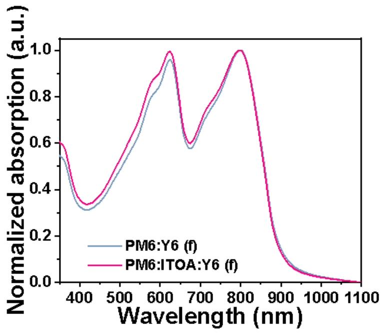

Figure S6. Normalized UV-vis absorption spectra of blend films.

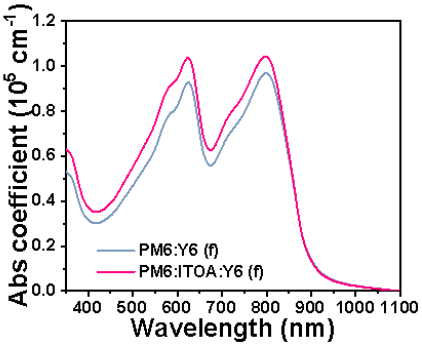

Figure S7 Absorption coefficient of neat PM6:Y6 and PM6:ITOA:Y6 blend films.

6. Cyclic voltammetry (CV) and Computational analyses

The electrochemical properties of the materials was measured by electrochemistry workstation (CHI660A, Chenhua Shanghai).

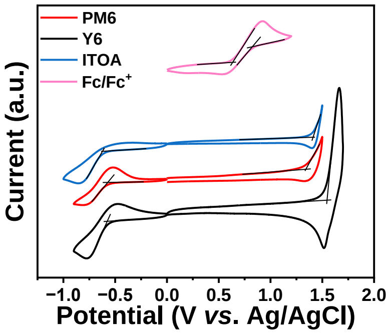

Figure S8 The results of cyclic voltammetry (CV) of PM6, Y6 and ITOA.

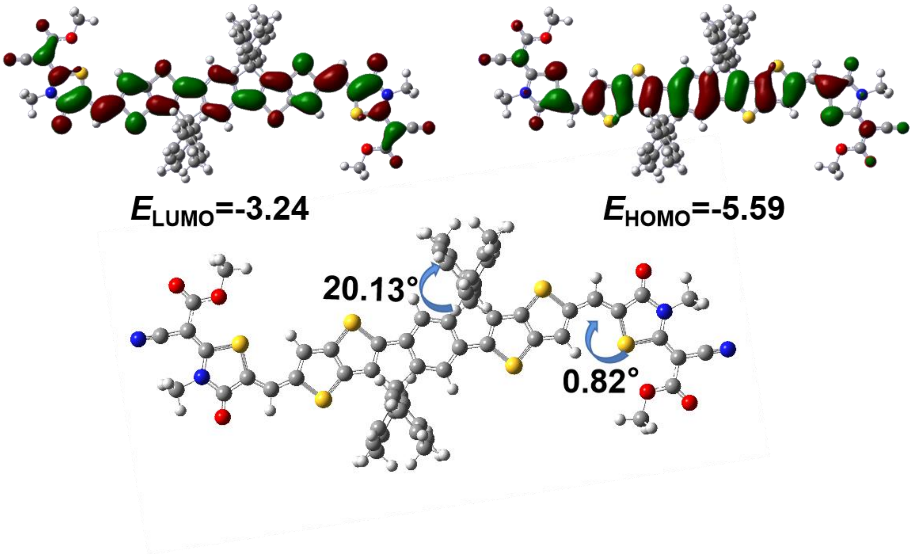

Figure S9 DFT of calculation at the B3LYP/631G (d, p) level of ITOA.

Table S1. Summary of optoelectronic properties of PM6, ITOA and Y6.

SM	λ_{abs}/film (nm)	λ_{onset}/film (nm)	$E_{opt}{}^{a}$ (eV)	$E_{HOMO}{}^{b}$ (eV)	$E_{LUMO}{}^{b}$ (eV)	E_{echem} (eV)	|HOMO|^{c} (eV)	|LUMO|^{c} (eV)	$HL_{gap}{}^{c}$ (eV)
ITOA	584	624	1.99	−5.48	−3.45	2.03	5.59	3.24	2.35

[a]Optical bandgaps estimated from the onset of the UV-Vis absorption spectra (films). [b]Estimated from cyclic voltammetry (CV) measurements; [c]DFT-calculated HOMO and LUMO energy levels (absolute values), and HOMO→LUMO gaps (HL_{gap}).

7. Device Fabrication

The solar cells were prepared on glass substrates with tin-doped indium oxide (ITO, 15 Ω sq^{-1}) patterned on the surface (device area: 0.1 cm^2). Substrates were first scrubbed with dilute Extran 300 detergent solution to remove organic residues before immersing in an ultrasonic bath of dilute Extran 300 for 15 min. Samples were rinsed in flowing deionized water for 5 min before being sonicated (Branson 5510) for 15 min each in successive baths of acetone and isopropanol. Next, the samples were exposed to a UV−ozone plasma for 30 min. The PEDOT:PSS solution was spin coated onto a cleaned ITO, subsequently annealed at 150℃ for 15 min in the air to obtain a PEDOT:PSS-covered ITO., and then transferred into a dry nitrogen glovebox (< 0.01 ppm O_2, < 0.01 ppm H_2O).

The chloroform solution (13.8 mg mL^{-1} in total) with 0.5 vol% of 1-CN was spin-coated on PEDOT:PSS layer with 2500 rpm for 30 s to obtain a photosensitive active layer. The active layers were spin-cast from the solutions using a programmable spin

coater from Specialty Coating Systems (Model G3P-8), resulting in films of ca. 100 nm in thickness. We chose PDINN as the electron buffer layer with 2500 rpm for 30 s. At last, the Ag electrode were slowly evaporated onto the surface of PDINN layer under a vacuum pressure of 5 × 10^{-6} Torr.

The *J-V* characteristics of the organic solar cells devices were measured by IVS-KA6000 Enlitech sunlight simulator equipped with an AM 1.5 filter at 100 mW cm^{-2} and Keithley SMU source after correcting the light intensity with a standard calibration cell. The corresponding EQE spectrum was acquired in air by a QE-R system from Enli Technology Co. Ltd.

Table S2 Device parameters of PM6:ITOA:Y6 (0.5%CN) with different weight ratio under thermal annealing at 90°C for 10 min.

Weight ratio	V_{OC} [V]	J_{SC} [mA cm^{-2}]	FF [%]	PCE [%]
1:0.05:1.2	0.870	27.57	77.63	18.62
1:0.1:1.2	0.871	27.49	75.94	18.18
1:0.2:1.2	0.873	27.36	72.49	17.42

Table S3 Device parameters of PM6:ITOA:Y6 (0.5%CN) with different thermal annealing temperature.[a]

Temperature [°C]	V_{OC} [V]	J_{SC} [mA cm^{-2}]	FF [%]	PCE [%]
80	0.872	27.30	76.14	18.13
90	0.870	27.57	77.63	18.62
100	0.865	27.67	76.34	18.27
110	0.862	27.81	75.81	18.17

[a] The ternary ratio is 1:0.05:1.2 for PM6:ITOA:Y6.

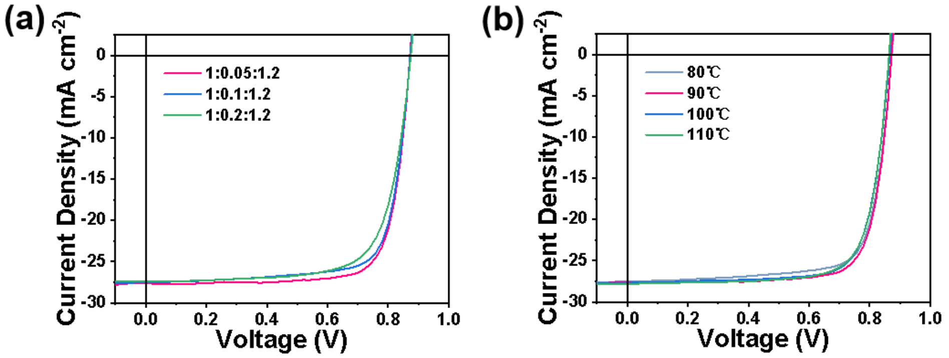

Figure S10 Current density-voltage (*J–V*) curves of PM6:ITOA:L8-BO with different (a) weight ratio and (b) thermal annealing temperature.

Table S4 Photovoltaic parameters of the reported PM6:Y6-based ternary OSCs with PCE over 18% and this work.

No.	Year	V_{OC} [V]	J_{SC} [mA cm^{-2}]	FF [%]	PCE [%]	Reference
1	2021	0.87	26.82	77.2	18.01	[1]

2	2022	0.846	27.92	77.13	18.21	[2]
3	2022	0.853	26.79	80.2	18.33	[3]
4	2022	0.87	27.68	75.36	18.15	[4]
5	2022	0.87	26.28	78.21	18.12	[5]
6	2022	0.87	26.65	77.85	18.1	[6]
7	2022	0.868	26.43	78.8	18.07	[7]
8	2022	0.863	27.38	76.46	18.07	[8]
9	2023	0.86	26.95	80.01	18.54	[9]
10	2023	0.88	27.17	77.26	18.41	[10]
11	2023	0.849	27.55	78.21	18.29	[11]
12	2023	0.848	27.47	77.2	18.0	[12]
13	2023	0.87	27.3	80.5	19.1	[13]
14	2023	0.870	27.57	77.63	18.62	This Work

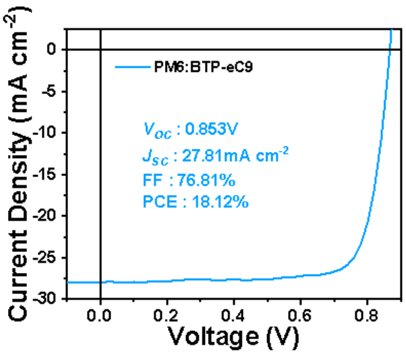

Figure S11 The *J-V* traces of PM6:BTP-eC9 binary OSCs.

Table S5 Photovoltaic parameters of the reported PM6:BTP-eC9-based ternary OSCs with PCE over 18.8% and this work.

No.	J_{SC}×FF	V_{OC} [V]	J_{SC} [mA cm^{-2}]	FF [%]	PCE [%]	Reference
1	21.68	0.87	27.2	79.7	18.8	[14]
2	21.78	0.866	27.05	80.51	18.86	[15]
3	22.00	0.862	27.09	81.2	18.96	[16]
4	21.95	0.866	27.3	80.4	19.0	[17]
5	22.02	0.8629	28.14	78.25	19.01	[18]
6	22.06	0.862	27.68	79.7	19.02	[19]
7	22.34	0.855	27.86	80.2	19.10	[20]
8	22.39	0.853	27.81	80.5	19.10	[21]
9	22.28	0.858	28.03	79.5	19.12	[22]
10	22.32	0.866	28.33	78.79	19.33	This Work

8. Energy Loss

Specification of the three sources of E_{loss} follows the equation:

E_{loss}= (E_g-qV_{oc}^{SQ})+q$V_{oc}^{rad,below\ gap}$+ q$V_{oc}^{non-rad}$= ΔE_1+ΔE_2+ΔE_3

where E_g is the band-gap, V_{oc}^{SQ} is the maximum V_{oc} under the S-Q limit, and V_{oc}^{rad} is the V_{oc} when only radiative recombination is considered. The final part of the non-radiative recombination loss (ΔE_3) is obtained by the calculation equation

ΔE_3= q$V_{oc}^{non-rad}$=$-kT\ln(EQE_{EL})$

where k is the Boltzmann constant, T is absolute temperature.

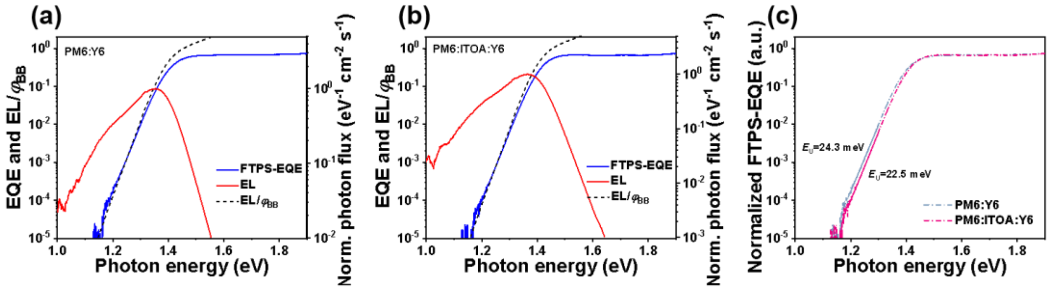

Figure S12 Normalized FTPS-EQE, EQE, and EL of the (a) PM6:Y6 and (b) PM6:ITOA:Y6 devices. (c) FTPS-EQE of PM6:Y6 and PM6:ITOA:Y6 devices at absorption onset.

9. SCLC Mobility

The hole and electron mobilities of BTC in optimized BHJ thin films were determined by fitting the dark current to the space-charge-limited current (SCLC) model using the following diode configuration: Glass/ITO/PEDOT:PSS/BHJ/MoO_3/Ag for hole-only diode, and Glass/ITO/ZnO/BHJ/PDINN/Ag for electron-only diode. The ITO substrates, bottom PEDOT:PSS layers and BHJ layers were prepared. A top MoO_3 layer (7.5 nm) was used as the electron-blocking layer and Silver cathode (100nm) were thermally evaporated through a shadow mask defining an active area of 0.04 cm^2 in the hole-only diodes. For electron-only device, the ZnO layer was spin coated by solution-processed method on top of ITO substrate. A PDINN layer (ca. 5nm) as the hole-blocking layer and Ag layer (100nm) as the anode were then thermally evaporated. Hole mobility and electron mobility were obtained by fitting the current density-voltage curves and calculated by the Eq. (1).

$$J(V)=\frac{9}{8}\varepsilon_0\varepsilon_r\mu_0\exp\left(0.89\beta\sqrt{\frac{V-V_{bi}}{L}}\right)\frac{(V-V_{bi})^2}{L^3} \quad (1)$$

Where J is current density, ε_0 is the permittivity of free space, ε_r is the relative permittivity of the material (assumed to 3), μ_0 is hole mobility or electron mobility, V is applied voltage, V_{bi} is the buit-in voltage (0 V), and L is the thickness of BHJ (ca. 100 nm).

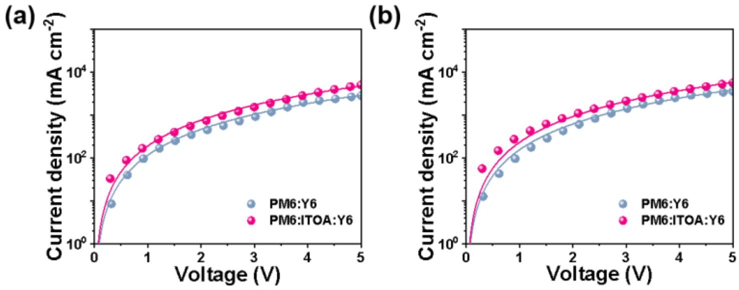

Figure S13 Dark current density-voltage characteristics at room temperature of optimized binary and ternary films for (a) hole-only diodes and (b) electron-only diodes. Note: The experimental data is fitted using the single-carrier SCLC model (solid lines). The solid lines are fits to the experimental data according to Equation.

Table S6 The carrier mobility of binary and ternary devices.

Active layer	Carrier mobility [×10^{-4}(cm^2 V^{-1} s^{-1})]		μ_h/μ_e
	μ_h	μ_e	
PM6:Y6	5.24	3.92	1.33
PM6:IDTR:Y6	7.84	6.45	1.21

10. Transient photovoltage (TPV) and transient photocurrent (TPC)

Transient photovoltage (TPV) and transient photocurrent (TPC) were characterized using Fluxim (PAIOS-4.0). TPV measurement was conducted under 1 sun conditions by illuminating the device with a white light-emitting diode, and the champion device was set to the open-circuit condition. For TPC measurement, the champion device was set to the short-circuit condition in dark. The output signal was collected by key sight oscilloscope. Voltages at open circuit and currents under short circuit conditions were measured over a 1 MΩ and a 50 Ω resistor, respectively.[23,24]

11. Grazing Incidence Wide-angle/Small-angle X-ray Scattering (GIWAXS/GISAXS)

GIWAXS and GISAXS was carried out on beamline BL16B1 at the Shanghai Synchrotron Radiation Facility (SSRF). The beam line has two detectors (PILATUS 900K for GIWAXS and PILATUS 2M detector for GISAXS, with a pixel size of 172 μm × 172 μm) installed in-line downstream of the sample to collect successively X-ray scattering data simultaneously. The distance from the sample to the detector set to 160 mm and 2000 mm. An incident photon energy of 10 keV was applied, with a

corresponding wavelength of 0.124 nm. An incident angle αi of 0.15° (near the critical angle) was applied in the GIWAXS/GISAXS experiment, thus providing a global and strong (averaged) scattering signal for the sample.[25,26] The GIWAXS/GISAXS data are collected with an exposure time of 30 s. One dimensional experimental data were obtained with the SGTools software package programmed by Zhao et al.[27]

Data analysis. The structural information of blend films such as the period of arrangement and lamellar stacking spacing is obtained via the Bragg equation, as well as the crystal coherence length (CCL) can be obtained from the Scherrer formula, and the specific expressions of the Bragg equation and Scherrer formula are as follows:[28]

$$d = \frac{\lambda}{2\sin(\theta)} = \frac{2\pi}{q} \tag{2}$$

$$CCL = \frac{K\lambda}{\mathrm{FWHM}\cdot\cos(\theta)} \tag{3}$$

where d is the lamellar stacking spacing, and CCL is the crystal domain along the specified direction called crystal coherence length, which is generally considered to be equivalent to the grain size. λ is the value of X-ray wavelength; K is a dimensionless shape factor, generally taken as $K = 0.89$; FWHM is the half-peak width of the scattering peak; θ is the scattering angle.[29]

Table S7 Detailed GIWAXS parameters of the binary and optimized ternary films.

	(010) diffraction peak				(100) diffraction peak			
	Q [nm^{-1}]	D [nm]	FWHM [nm^{-1}]	CCL [nm]	Q [nm^{-1}]	D [nm]	FWHM [nm^{-1}]	CCL [nm]
PM6:Y6	18.64	0.34	3.69	1.70	3.17	1.98	1.06	5.92
PM6:ITOA:Y6	18.68	0.34	3.53	1.78	3.29	1.91	0.99	6.34

GISAXS fitting model. To quantify and compare the phase separation, the IP (in plane) scattering profiles are fitted with a model that describes the scattering contribution of each phase, The fitting equation is shown in Eq. (4):

$$I(q) = \frac{A_1}{[1+(q\xi)^2]^2} + A_2\langle P(q,R)\rangle S(q,R,\eta,D) + B \tag{4}$$

where the first term was the so-called Debye-Anderson-Brumberger (DAB) term, modeling the scattering from the amorphous intermixing domains, and ξ is the average correlation length of the amorphous domain.

12. Contact Angles (CA)

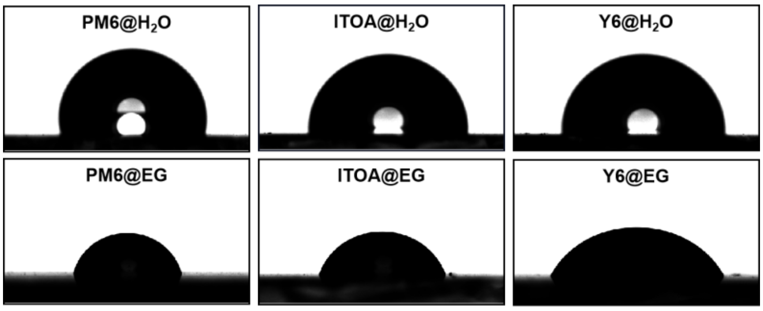

Figure S14 The contact angles of water and ethylene glycol drops on PM6, ITOA and Y6 films.

Table S8 Summary of contact angles and interaction parameters for PM6, ITOA and Y6.

Material	Water contact angle [°]	EG contact angle [°]	γ^d [mN m^{-1}]	γ^p [mN m^{-1}]	Surface tension [mN m^{-1}]	$\chi_{\mathrm{PM6:ITOA}}$ [K]	$\chi_{\mathrm{Y6:ITOA}}$ [K]
PM6	103.67	75.47	26.51	0.28	26.79	—	—
ITOA	90.46	71.32	14.48	6.60	21.08	0.34	0.91
Y6	91.86	62.49	28.73	2.04	30.77	—	—

γ^d and γ^p represent the dispersion component and polar component of surface free energy, respectively, γ is the surface free energy, $\gamma = \gamma^d + \gamma^p$.

13. Film-depth-dependent light absorption spectroscopy (FLAS)

A self-developed soft plasma source generated by glow-discharging of low-pressure oxygen was used to incrementally etch the film without degage to the underneath films, which were in-situ monitored by a spectrometer. FLAS is extracted from the evolution of the light absorption spectra during soft plasma etching. The film-depth-dependent exciton generation is obtained upon the modified optical transmission matrix method, taking film-depth-dependent light absorption spectra and optical interference into simulation. The detail of the optical modeling is available in the literature published by Lu et a1.[30]

14. In-situ UV-vis absorbance during the spin-coating process

According to the preparation process of the devices, the chloroform solution (13.8 mg mL^{-1} in total) with 0.5 vol% of 1-CN was spin-coated on clean quartz flakes with 2500 rpm for 30 s. In this process, the in-situ light absorption spectra, fluorescence spectra and light scattering spectra were performed on a multi-spectrometer (DU-200, Shaanxi Puguang Weishi Co. Ltd.).

15. Additional PV Device Performance Data

Table S9 Photovoltaic parameters of the reported no post-processing devices with PCE over 15% OSCs and this work.

No.	Year	V_{OC} [V]	J_{SC} [mA cm^{-2}]	FF [%]	PCE [%]	Reference

1	2022	0.86	24.67	71.35	15.2	[31]
2	2023	0.852	25.3	70.7	15.2	[32]
3	2022	0.85	24.13	74.35	15.27	[33]
4	2020	0.854	24.27	74	15.34	[34]
5	2022	0.86	24.5	76.5	16.1	[35]
6	2022	0.877	25.86	71.85	16.39	[36]
7	2021	0.896	24.86	74.8	16.68	[37]
8	2022	0.86	26.36	74.45	16.87	[38]
9	2022	0.846	26.44	77.42	17.33	[39]
10	2023	0.87	26.88	75.35	17.62	[40]
11	2020	0.839	27.98	75.3	17.7	[41]
12	2020	0.900	25.87	76.92	17.91	[42]
13	2023	0.874	27.37	75.46	18.04	This Work

16. Reference

[1] F. Liu, L. Zhou, W. Liu, Z. Zhou, Q. Yue, W. Zheng, R. Sun, W. Liu, S. Xu, H. Fan, L. Feng, Y. Yi, W. Zhang, X. Zhu, *Adv. Mater.* **2021**, *33*, 2100830.

[2] S. Zhang, F. Bi, J. Han, C. Shang, X. Kang, X. Bao, *Nano Energy* **2022**, *102*, 107742.

[3] D. Li, F. Geng, T. Hao, Z. Chen, H. Wu, Z. Ma, Q. Xue, L. Lin, R. Huang, S. Leng, B. Hu, X. Liu, J. Wang, H. Zhu, M. Lv, L. Ding, M. Fahlman, Q. Bao, Y. Li, *Nano Energy* **2022**, *96*, 107133.

[4] P. Wang, Y. Li, C. Han, J. Wang, F. Bi, N. Zheng, J. Yang, J. Wang, X. Bao, *J. Mater. Chem. A* **2022**, *10*, 17808–17816.

[5] S. Zhang, X. Ma, C. Xu, W. Xu, S. Y. Jeong, H. Y. Woo, Z. Zhou, X. Zhang, F. Zhang, *Macromol. Rapid Commun.* **2022**, *43*, 2200345.

[6] H. Tan, B. Yuan, Z. Shao, W. Deng, J. Yu, M. Xiao, H. Wu, W. Zhu, *Chem. Eng. J.* **2022**, *445*, 136691.

[7] W. Feng, S. Wu, H. Chen, L. Meng, F. Huang, H. Liang, J. Zhang, Z. Wei, X. Wan, C. Li, Z. Yao, Y. Chen, *Adv. Energy Mater.* **2022**, *12*, 2104060.

[8] M. Guan, W. Tao, L. Xu, Y. Qin, J. Zhang, S. Tan, M. Huang, B. Zhao, *J. Mater. Chem. A* **2022**, *10*, 9746–9752.

[9] C. Zhang, M. Zhang, Q. Zhou, S. Chen, S. Kim, J. Yao, Z. Zhang, Y. Bai, Q. Chen, B. Chang, H. Fu, L. Xue, H. Wang, C. Yang, Z. Zhang, *Adv. Funct. Mater.* **2023**, *33*, DOI 10.1002/adfm.202214392.

[10] F. Qi, Y. Li, R. Zhang, F. R. Lin, K. Liu, Q. Fan, A. K. -Y. Jen, *Angew. Chemie Int. Ed.* **2023**, *62*, 2303066.

[11] M. Liu, X. Ge, X. Jiang, F. Guo, S. Gao, Q. Peng, L. Zhao, Y. Zhang, *Adv. Funct. Mater.* **2023**, *33*, 2300214.

[12] M. Liu, X. Ge, X. Jiang, D. Chen, F. Guo, S. Gao, Q. Peng, L. Zhao, Y. Zhang, *Nano Energy* **2023**, *112*, 108501.

[13] X. Li, A. Tang, H. Wang, Z. Wang, M. Du, Q. Guo, Q. Guo, E. Zhou, *Angew. Chemie Int. Ed.* **2023**, *62*, 2306847.

[14] J. Wang, M. Zhang, J. Lin, Z. Zheng, L. Zhu, P. Bi, H. Liang, X. Guo, J. Wu, Y. Wang, L. Yu, J. Li, J. Lv, X. Liu, F. Liu, J. Hou, Y. Li, *Energy Environ. Sci.* **2022**, *15*, 1585–1593.
[15] P. Bi, S. Zhang, Z. Chen, Y. Xu, Y. Cui, T. Zhang, J. Ren, J. Qin, L. Hong, X. Hao, J. Hou, *Joule* **2021**, *5*, 2408–2419.
[16] R. Ma, C. Yan, P. W.-K. Fong, J. Yu, H. Liu, J. Yin, J. Huang, X. Lu, H. Yan, G. Li, *Energy Environ. Sci.* **2022**, *15*, 2479–2488.
[17] H. Chen, S. Y. Jeong, J. Tian, Y. Zhang, D. R. Naphade, M. Alsufyani, W. Zhang, S. Griggs, H. Hu, S. Barlow, H. Y. Woo, S. R. Marder, T. D. Anthopoulos, I. McCulloch, Y. Lin, *Energy Environ. Sci.* **2023**, *16*, 1062–1070.
[18] C. Zhu, J. Tian, W. Liu, Y. Duan, Y. Song, Z. You, X. Wang, N. Li, X. Zhan, T. P. Russell, Y. Liu, *ACS Energy Lett.* **2023**, *8*, 2689–2698.
[19] H. Gao, R. Yu, Y. Gong, Z. Ma, Z. He, Y. Dong, K. Xu, Y. Bai, Z. Tan, *Small* **2022**, *18*, 2205128.
[20] M. Zhou, C. Liao, Y. Duan, X. Xu, L. Yu, R. Li, Q. Peng, *Adv. Mater.* **2023**, *35*, 2208279.
[21] R. Ma, X. Jiang, J. Fu, T. Zhu, C. Yan, K. Wu, P. Müller-Buschbaum, G. Li, *Energy Environ. Sci.* **2023**, *16*, 2316–2326.
[22] C. Han, J. Wang, S. Zhang, L. Chen, F. Bi, J. Wang, C. Yang, P. Wang, Y. Li, X. Bao, *Adv. Mater.* **2023**, *35*, 2208986.
[23] C. Yang, M. Jiang, S. Wang, B. Zhang, P. Mao, H. Y. Woo, F. Zhang, J. Wang, Q. An, *Adv. Mater.* **2024**, *36*, 2305356.
[24] H. Bai, Q. An, M. Jiang, H. S. Ryu, J. Yang, X. Zhou, H. Zhi, C. Yang, X. Li, H. Y. Woo, J. Wang, *Adv. Funct. Mater.* **2022**, *32*, 2200807.
[25] X. Sun, K. Liu, N. Zhao, F. Bian, C. Yang, Y. Huang, *J. Phys. Chem. B* **2022**, *126*, 1625–1632.
[26] X. Sun, W. Huang, C. Yang, F. Qi, J. Chen, N. Zhao, X. OuYang, *ACS Appl. Energy Mater.* **2023**, *6*, 6826–6833.
[27] N. Zhao, C. Yang, F. Bian, D. Guo, X. Ouyang, *J. Appl. Crystallogr.* **2022**, *55*, 195–203.
[28] A. Mahmood, J. Wang, *Sol. RRL* **2020**, *4*, 2000337.
[29] D.-M. Smilgies, *J. Appl. Crystallogr.* **2009**, *42*, 1030–1034.
[30] L. Bu, M. Hu, W. Lu, Z. Wang, G. Lu, *Adv. Mater.* **2018**, *30*, 1704695.
[31] D. Li, H. Lu, Y.-N. Chen, X. Ma, H. Zhang, H. Wang, X. Yu, X. Xu, Z. Zhang, X. Xu, Y. Liu, Z. Ma, A. Zhang, Z. Bo, *Chem. Mater.* **2022**, *34*, 8840–8848.
[32] H. Liu, Y. Fu, Z. Chen, J. Wang, J. Fu, Y. Li, G. Cai, C. Su, U. Jeng, H. Zhu, G. Li, X. Lu, *Adv. Funct. Mater.* **2023**, *33*, 2303307.
[33] L. Zhong, S. Kang, J. Oh, S. Jung, Y. Cho, G. Park, S. Lee, S. Yoon, H. Park, C. Yang, *Adv. Funct. Mater.* **2022**, *32*, 2201080.
[34] J. Zhang, Y. Han, W. Zhang, J. Ge, L. Xie, Z. Xia, W. Song, D. Yang, X. Zhang, Z. Ge, *ACS Appl. Mater. Interfaces* **2020**, *12*, 57271–57280.
[35] J. S. Park, C. Sun, Y. Han, G.-U. Kim, T. N.-L. Phan, Y.-H. Kim, B. J. Kim, *Adv. Energy Sustain. Res.* **2022**, *3*, 2200070.
[36] S. Lee, G. Park, M. Jeong, B. Lee, S. Jeong, J. Park, Y. Cho, S. M. Noh, C. Yang,

ACS Appl. Mater. Interfaces **2022**, *14*, 33614–33625.

[37] R. Ma, Y. Tao, Y. Chen, T. Liu, Z. Luo, Y. Guo, Y. Xiao, J. Fang, G. Zhang, X. Li, X. Guo, Y. Yi, M. Zhang, X. Lu, Y. Li, H. Yan, *Sci. China Chem.* **2021**, *64*, 581–589.

[38] W. Sun, H. Chen, B. Zhang, Q. Cheng, H. Yang, Z. Chen, G. Zeng, J. Ding, W. Chen, Y. Li, *Chinese J. Chem.* **2022**, *40*, 2963–2972.

[39] X. Xu, Y. Qi, X. Luo, X. Xia, X. Lu, J. Yuan, Y. Zhou, Y. Zou, *Fundam. Res.* **2023**, *3*, 611–617.

[40] X. Ma, W. Xu, Z. Liu, S. Y. Jeong, C. Xu, J. Zhang, H. Y. Woo, Z. Zhou, F. Zhang, *ACS Appl. Mater. Interfaces* **2023**, *15*, 7247–7254.

[41] Z. Zhang, Y. Li, G. Cai, Y. Zhang, X. Lu, Y. Lin, *J. Am. Chem. Soc.* **2020**, *142*, 18741–18745.

[42] X. Ma, A. Zeng, J. Gao, Z. Hu, C. Xu, J. H. Son, S. Y. Jeong, C. Zhang, M. Li, K. Wang, H. Yan, Z. Ma, Y. Wang, H. Y. Woo, F. Zhang, *Natl. Sci. Rev.* **2021**, *8*, DOI 10.1093/nsr/nwaa305.
